# Cases of Inflammation of the Absorbents, Cured by the Application of the Lunar Caustic

**Published:** 1826-09

**Authors:** John Higginbottom


### INFLAMMATION OF THE ABSORBENTS.

*Cases of Inflammation of the Absorbents, cured by the Application of the Lunar Caustic.* By John Higginbottom, Esq.

It is my intention to request the favour of you to insert in your Journal such observations as I may make, from time to time, in my investigation into the efficacy of the application of the lunar caustic. In my first communication, in your Number for May, I described the effects of the lunar caustic in certain cases of local inflammation. I have now the pleasure of sending you an important application of this remedy for the cure of that most painful and alarming affection, *inflammation of the absorbents.*

Case I.—Mrs. H—, aged thirty-four, had a swelling similar to a boil on the fore-arm near the wrist, the centre of which had a vesicular appearance; and, on removing the loose skin, presented a small ulcer, with highly inflamed and irregular edges, surrounded by inflammation. The absorbents were inflamed on the inside of the forearm, nearly to the axilla. Mrs. H— could assign no cause for this affection, which had been coming on for four or five days. She complained of feeling unwell and feverish.

The lunar caustic was applied to the ulcer, and slightly over the surrounding inflammation, along the course of the inflamed absorbents, and on the surrounding skin wherever there was any swelling. A purgative, with blue-pill and infusion of senna and salts, were administered.

On the following day, the inflammation was observed to be completely checked in its progress. The patient reported, that, in about half an hour after the application of the caustic, she experienced sensible amendment; that the arm became much cooler and easier, and it remained so during the night.

On the second morning, there was increased heat of the parts where the caustic had been applied, and slight vesications on the parts which had been before most inflamed,—viz. along the absorbents and around the ulcerated part. The vesicles were opened with a needle, and a puriform fluid was evacuated; and afterwards the parts were exposed to the air to dry.

On the third day, there was vesication nearly all over the parts which were previously inflamed; but on the fourth day no vesication remained: the cuticle was peeling off, leaving the parts free from inflammation. The eschar appeared to be adherent to the ulcerated part.

On the fifth day, there was a slight discharge from under the eschar on the ulcerated part, which continued for several days; afterwards the eschar became adherent. From this time no further attention was required.

Case II.—Ann Shipham, aged eleven years, had a fall upon the elbow, about a fortnight ago, which caused a wound about the size of a sixpence: it was neglected till it began to inflame, when her parents dressed it with some sticking-plaster, which produced increased inflammation.

At the time she applied to me, the sore on the elbow was very much inflamed and irritable, and surrounded with inflammation to the extent of an inch and a half; the absorbents were inflamed, and presented well-marked red lines up to the axilla, where there was an enlargement of the glands, attended with great tenderness. The patient complained of shivering, the tongue was furred, and there were headache and loss of appetite.

I wished to try the effect of the caustic without using any other means whatever, so that I ordered her no medicine. I applied the caustic along the inflamed absorbents, over the tumor in the axilla, and slightly over the wound and the surrounding inflamed part. I then applied gold-beater's skin over the ulcer; the other parts were exposed to the air, and directed to be kept free from covering.

On the following morning, I learnt from my patient that she had had a restless night, and some smarting pain; her countenance, however, was improved; and, though the tongue still remained furred, she expressed herself as being much better, having had no headache. There was some vesication on the parts most inflamed, with some tenderness, but no other pain; that along the inflamed absorbents and in the axilla having entirely subsided. I made no opening into the vesications.

On the second morning, she made no complaint: the tongue was quite clean; there was no tenderness on pressure in the axilla, nor any fluid under the vesication, except a little in the most dependent part near the elbow. The cuticle above was shrinking; the eschar remained adherent on the wound from the fresh application of the caustic.

On the third morning, she appeared quite well.

On the fourth morning, the tongue had become white, and I directed a little opening medicine. The arm remained perfectly well.

I might not have sent these two cases, but for the reasons already given in my former paper,—viz. that others may be induced to make similar trials; and for the fact that such cases are not of very frequent occurrence, so that much time might be lost by the delay of publication.

For the same reasons, too, I here add a case of ***diffused phlegmonous inflammation,*** equally successfully treated by the caustic, and perhaps not of less interest than those just given.

Case III.—Miss ——, aged fourteen, was brought to me with considerable swelling, hardness, and redness of the skin, seated in the ham, and extending along the back part of the thigh and the calf of the leg, which had been increasing for four days. No cause could be assigned for this affection. The patient was feverish, and complained of feeling generally indisposed. The inflammation had a phlegmonous character.

I applied the lunar caustic lightly over the whole surface of the inflammation, the part being previously moistened with water.

It may be necessary to note here, that I always apply the caustic in the solid form, and find a slight application sufficient, if it passes over every part of the inflamed surface. On the inside of the hand and of the feet, where the cuticle is thick, a freer application may be made, as vesication does not so readily take place; but even in these parts I have not found it necessary to use it very freely. I always remove any moisture which may be left upon the part with a little linen or lint, so as to prevent its running upon any neighbouring parts.

I had directed my patient to see me the following day, but three days having elapsed without hearing from her, I sent to her, and was informed by her mother that she did not think it necessary to come again, as the patient was nearly well. On seeing her, I found no vestige of inflammation or swelling. A slight vesication had been caused the day after the application of the caustic, for which the mother had applied a little simple ointment; the cuticle was separating in some parts, leaving the surface white. My patient had experienced no pain from the application of the caustic, and only a little heat, occasioned by the vesications.

In five days, the cuticle had nearly all separated, and the patient was quite well.

I would here observe, that I have no idea that the two first of these cases could have been so cut short by any other known means; or that the third could possibly have been prevented from ending in suppuration. I have stated these effects thus strongly, but simply, in order that a remedy so manifestly important may not be overlooked.

My views respecting the efficacy of the application of the lunar caustic in certain cases of inflammatory affections, seem to derive great support from the observations of Baron Larrey relative to the effects of the actual cautery; an account of which you have given in your Number for April.

*Nottingham; June 6th,* 1826.

